# Identification of *Burkholderia mallei *and *Burkholderia pseudomallei *adhesins for human respiratory epithelial cells

**DOI:** 10.1186/1471-2180-10-250

**Published:** 2010-09-28

**Authors:** Rachel Balder, Serena Lipski, John J Lazarus, William Grose, Ronald M Wooten, Robert J Hogan, Donald E Woods, Eric R Lafontaine

**Affiliations:** 1 Department of Infectious Diseases, University of Georgia College of Veterinary Medicine, Athens, GA 30602, USA; 2 Department of Medical Microbiology and Immunology, University of Toledo Health Sciences Campus, 3055 Arlington Avenue, Toledo, OH 43614, USA; 3 Department of Microbiology and Infectious Diseases, University of Calgary Health Sciences Centre, 3330 Hospital Drive, NW Calgary, Alberta T2N 4N1, Canada

## Abstract

**Background:**

*Burkholderia pseudomallei *and *Burkholderia mallei *cause the diseases melioidosis and glanders, respectively. A well-studied aspect of pathogenesis by these closely-related bacteria is their ability to invade and multiply within eukaryotic cells. In contrast, the means by which *B. pseudomallei *and *B. mallei *adhere to cells are poorly defined. The purpose of this study was to identify adherence factors expressed by these organisms.

**Results:**

Comparative sequence analyses identified a gene product in the published genome of *B. mallei *strain ATCC23344 (locus # BMAA0649) that resembles the well-characterized *Yersinia enterocolitica *autotransporter adhesin YadA. The gene encoding this *B. mallei *protein, designated *boaA*, was expressed in *Escherichia coli *and shown to significantly increase adherence to human epithelial cell lines, specifically HEp2 (laryngeal cells) and A549 (type II pneumocytes), as well as to cultures of normal human bronchial epithelium (NHBE). Consistent with these findings, disruption of the *boaA *gene in *B. mallei *ATCC23344 reduced adherence to all three cell types by ~50%. The genomes of the *B. pseudomallei *strains K96243 and DD503 were also found to contain *boaA *and inactivation of the gene in DD503 considerably decreased binding to monolayers of HEp2 and A549 cells and to NHBE cultures.

A second YadA-like gene product highly similar to BoaA (65% identity) was identified in the published genomic sequence of *B. pseudomallei *strain K96243 (locus # BPSL1705). The gene specifying this protein, termed *boaB*, appears to be *B. pseudomallei*-specific. Quantitative attachment assays demonstrated that recombinant *E. coli *expressing BoaB displayed greater binding to A549 pneumocytes, HEp2 cells and NHBE cultures. Moreover, a *boaB *mutant of *B. pseudomallei *DD503 showed decreased adherence to these respiratory cells. Additionally, a *B. pseudomallei *strain lacking expression of both *boaA *and *boaB *was impaired in its ability to thrive inside J774A.1 murine macrophages, suggesting a possible role for these proteins in survival within professional phagocytic cells.

**Conclusions:**

The *boaA *and *boaB *genes specify adhesins that mediate adherence to epithelial cells of the human respiratory tract. The *boaA *gene product is shared by *B. pseudomallei *and *B. mallei *whereas BoaB appears to be a *B. pseudomallei*-specific adherence factor.

## Background

*Burkholderia pseudomallei *is a Gram-negative bacterium readily recovered from the water and wet soils of endemic areas bordering the equator, particularly Southeast Asia and Northern Australia [1-9]. The organism is a motile, aerobic bacillus that can survive environmental extremes as well as the bactericidal activities of complement [10-12], defensins [13-15], and phagocytes [1,2,16-18]. The genome of the *B. pseudomallei *isolate K96243 has been published by the Wellcome Trust Sanger Institute and was shown to consist of two chromosomes of 4.1 and 3.2 Mbp [19]. *Burkholderia mallei *is a non-motile, host-adapted clone of *B. pseudomallei *that does not persist outside of its equine host and is endemic to certain parts of Asia, Africa, the Middle East and South America [8,9,20-25]. The genomic sequence of the *B. mallei *strain ATCC23344 has been published by TIGR [26] and is smaller (2 chromosomes of 3.5 and 2.3 Mbp) than that of *B. pseudomallei *K96243. *B. mallei *ATCC23344 was found to specify a large number of mobile DNA elements that have contributed to extensive deletions and rearrangements relative to the genome of *B. pseudomallei *K96243. Despite these differences, the genes shared by the two isolates have an average identity of 99% at the nucleotide level [19,26]. The genomic sequence of several *B. pseudomallei *and *B. mallei *isolates are also publicly available through the NCBI genomic BLAST service (http://www.ncbi.nlm.nih.gov/sutils/genom_table.cgi), which provides a wealth of resources to study these organisms.

*B. pseudomallei *causes the human disease melioidosis, which is notoriously difficult to diagnose. Clinical manifestations vary greatly and may present as flu-like symptoms, benign pneumonitis, acute and chronic pneumonia, or fulminating septicemia. Infection occurs via inhalation of contaminated aerosol particles or through skin abrasions, and the risk of contracting the disease is proportional to the concentration of *B. pseudomallei *in soil and water. In endemic areas, heavy rainfalls result in a shift from percutaneous inoculation to inhalation as the primary mode of infection, which leads to a more severe illness. Melioidosis commonly affects the lungs and is characterized by the spread of bacteria to various internal organs including the spleen and liver. Many patients become bacteremic and the mortality rate is high (19-51%) despite aggressive antimicrobial therapy [1-9]. *B. pseudomallei *is refractory to most antibiotics and resistance mechanisms include efflux pumps and β-lactamases [27-36]. The recommended treatment entails the use of ceftazidime, carbapenems, TMP-SMZ, chloramphenicol and/or Augmentin for several weeks. Response to treatment is slow and eradication of *B. pseudomallei *is difficult to achieve, resulting in recrudescence [1,37-39].

*B. mallei *causes the zoonosis glanders, which primarily affects solipeds [8,9,20-25]. In humans, infection occurs by contact with infected animals via the cutaneous or respiratory route. The clinical manifestations of the disease include febrile pneumonia associated with necrosis of the tracheobronchial tree or pustular skin lesions and the development of abscesses. Most patients become bacteremic and *B. mallei *disseminates to the liver and spleen where it rapidly causes necrosis. Even with antibiotic treatment, the mortality rate for human glanders is 50% and the basis for this high mortality rate is not understood, though *B. mallei *has been shown to be resistant to complement-mediated killing [40], macrophages [41] and antimicrobials [32,42].

One key aspect of pathogenesis by *B. mallei *and *B. pseudomallei *is their ability to invade and multiply within a variety of eukaryotic cells, where bacteria are shielded from the host humoral immune response and antibiotics. Once internalized, *B. mallei *and *B. pseudomallei *escape from endocytic vacuoles and enter the cytoplasm of infected cells where they multiply. The organisms subsequently spread to neighboring cells through a process involving the formation of actin tails and membrane protrusions that push bacteria from one cell to another. This intracellular lifestyle is crucial to virulence and has been a focus of research efforts aimed at understanding pathogenesis by *B. mallei *and *B. pseudomallei *[2,9,16-18,22,41,43-49]. Several gene products, such as BimA, type 3 secretion system effectors, and type 6 secretion proteins, have been shown to play key roles in this process. By contrast, the mechanisms used by these organisms to adhere to eukaryotic cells are poorly defined. Adherence is an essential step of pathogenesis by most infectious agents because it is necessary for colonizing a new host [50-52]. Moreover, *B. pseudomallei *and *B. mallei *are facultative intracellular pathogens that gain access to the interior of target cells. Though not always a prerequisite for this process, bacterial adherence is a widespread strategy that precedes and promotes invasion [50-52]. Thus far, only the *B. pseudomallei *flagellum [53] and type 4 pilus [54] have been implicated in adherence and their exact roles remain to be elucidated. The present study reports the identification of *B. pseudomallei *and *B. mallei *gene products that mediate adherence to epithelial cells derived from the human respiratory tract, thus relevant to the aerosol route of infection by these organisms.

## Results

### Identification of a gene shared by *B. mallei *and *B. pseudomallei *that encodes a potential autotransporter adhesin

**Figure 1:**
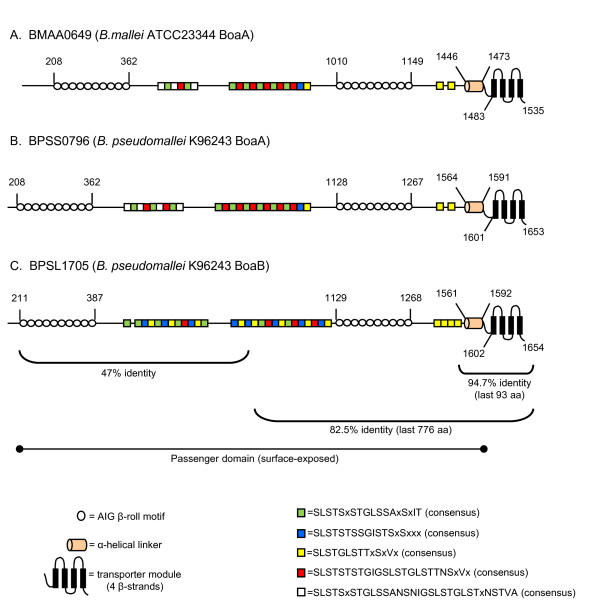
**Structural features of the *boaA *and *boaB *gene products**. Different regions of the predicted *B. mallei *ATCC23344 BoaA (A), *B. pseudomallei *K96243 BoaA (B) and *B. pseudomallei *K96243 BoaB (C) proteins are depicted with the positions of residues defining selected domains. The horizontal brackets outline selected regions of the BoaA and BoaB proteins and the percent identity between these regions is shown below the brackets. Transporter modules (OM anchors) and helical linkers were identified using the PSIPRED secondary structure prediction algorithm. The colored boxes show the relative position and number of repeated SLST motifs.

Analysis of the annotated genomic sequence of *B. mallei *ATCC23344 identified the ORF locus tag number BMAA0649 as resembling members of the oligomeric coiled-coil adhesin (Oca) family of autotransporter proteins [55]. *Yersinia enterocolitica *YadA [55-57] is the prototypical member of this group of adherence factors, which also includes *Haemophilus influenzae *Hia [58-60] and *Moraxella catarrhalis *Hag [61,62]. These Oca proteins share structural features including a C-terminal outer membrane (OM) anchor domain composed of 4 β-strands (also referred to as the transporter module), a surface-exposed passenger domain often containing repeated amino acid (aa) motifs, and a helical region of ~40 residues that connects the OM anchor to the surface-exposed passenger domain [55,63-65]. As illustrated in Fig 1A, BMAA0649 is predicted to possess these features. Further sequence analysis of the *B. mallei *ATCC23344 gene product revealed that residues 208-362 (and 1010-1149) contain repeats with the consensus xxxAV**AIG**xx[N/A]xAx (open circles in Fig 1A), which resemble motifs found in the N-terminus of *Y. enterocolitica *YadA (xxxSV**AIG**xxSxAx) [56,57] and *M. catarrhalis *Hag (GxxSI**AIG**xx[A/S]xAx) [61]. In YadA, these AIG patterns have been shown to form a structure termed a β-roll and to specify adhesive properties. The passenger domain of BMAA0649 was also found to contain several serine-rich repeats beginning with residues SLST (colored squares in Fig 1A). Additionally, searches using the Pfam database indicated that aa 1456-1535 of BMAA0649 encode a YadA-like C-terminal domain (PF03895; expect value 3.8 e^-11^), which is present in most Oca molecules and is described as important for oligomerization. Taken together, these observations suggest structural and functional similarities between BMAA0649 and members of the Oca family of autotransporters. Hence, we designated this ORF of *B. mallei *ATCC23344 *boaA *(*Burkholderia O*ca-like *a*dhesin *A***)**. Table 1 lists characteristics of the *boaA *gene and its encoded product.

**Table 1:** Characteristics^a ^of *boaA *and *boaB *genes and their encoded products

Strain	Gene	Chromosome	Locus tag	GenBank accession #	ORF (nt)	Predicted protein (aa)	MW (Da)	Potential signal sequence **cleavage site**^**b**^
*B.mallei*								
ATCC23344	*boaA*	2	BMAA0649	YP_105401.1	4608	1535	140,689	WA^18^▼GV
NCTC10247	*boaA*	2	BMA10247_A1776	YP_001078959.1	5301	1766	162,744	WA^77^▼GV
*B. pseudomallei*								
K96243	*boaA*	2	BPSS0796	YP_110805.1	4962	1653	151,565	WA^18^▼GV
DD503	*boaA*	ND	-	EF423807	4680	1559	143,209	WA^18^▼AL
1710b	*boaA*	2	BURPS1710b_A2381	YP_337531.1	4881	1626	149,383	WA^10^▼AL

K96243	*boaB*	1	BPSL1705	YP_108306.1	4821	1606	148,811	VA^23^▼GT
DD503	*boaB*	ND	-	EF423808	4965	1654	154,117	VA^71^▼GT
1710b	*boaB*	1	BURPS1710b_2168	YP_333563.1	4965	1654	154,059	VA^71^▼GT

^a^Sequence analyses were performed using Vector NTI (Invitrogen) and online tools available through the ExPASy Proteomics Server.

^b^The putative signal sequence cleavage site was determined using the SignalP 3.0 server

ND = not determined

The published genome of *B. pseudomallei *K96243 was also found to specify a *boaA *gene product (BPSS0796, Fig 1B) that is 92.7% identical to that of *B. mallei *ATCC23344. Oligonucleotide primers were designed to amplify the entire *boaA *gene from the *B. pseudomallei *strain used in our laboratory, DD503, and sequence analysis of this amplicon predicted a gene product that is 94.4% and 90.6% identical to BoaA of *B. mallei *ATCC23344 and *B. pseudomallei *K96243, respectively. Database searches with the NCBI genomic BLAST service also identified *boaA *in several *B. pseudomallei *and *B. mallei *isolates. All nine *B. mallei *and 23 *B. pseudomallei *strains for which sequences are available through this service were found to have the gene. Characteristics of some of these ORFs are listed in Tables 1 and 2. Overall, the BoaA proteins are 82-94% identical and differ primarily in the number and/or arrangement of SLST repeats in their predicted passenger domains (data not shown). Based on these results, we conclude that BoaA is a well-conserved gene product shared by *B. mallei *and *B. pseudomallei*.

**Table 2:** Percent identity shared by *boaA *and *boaB *gene products

	BoaA (*Bm *ATCC23344)	BoaA (*Bm *NCTC10247)	BoaA (*Bp *K96243)	BoaA (*Bp *DD503)	BoaA (*Bp *1710b)	BoaB (*Bp *K96243)	BoaB (*Bp *DD503)	BoaB (*Bp *1710b)
BoaA (*Bm *ATCC23344)	100							
BoaA (*Bm *NCTC10247)	86.9	100						
BoaA (*Bp *K96243)	92.7	89.2	100					
BoaA (*Bp *DD503)	94.4	82.2	90.6	100				
BoaA (*Bp *1710b)	90.4	83.1	92.4	93.6	100			

BoaB (*Bp *K96243)	64	60	65	63.9	63.9	100		
BoaB (*Bp *DD503)	62	60.8	62.9	61.9	62.2	96.7	100	
BoaB (*Bp *1710b)	62.2	60.9	63.2	62.1	62.4	97	99.7	100

*Bm *= *B. mallei*

*Bp *= *B. pseudomallei*

### Identification of a *B. pseudomallei*-specific gene encoding a putative autotransporter adhesin that resembles BoaA

**Figure 2:**
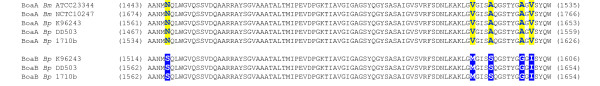
**Sequence comparison of *boaA *and *boaB *gene products**. The last 93 residues of selected *boaA *and *boaB *gene products are shown with the positions of the aa defining these regions in parentheses. Perfectly conserved aa are shown in black text over white background. Residues unique to BoaA proteins are shown in blue text over a yellow background. Residues unique to BoaB proteins are shown in white text over a blue background. *Bm *= *B. mallei*, *Bp *= *B. pseudomallei*.

Further analysis of the annotated genomic sequence of *B. pseudomallei *K96243 identified the ORF locus tag number BPSL1705 as specifying a second Oca-like protein that is ~60% identical to BoaA. The last 776 aa of BPSL1705 and BoaA are 82.5% identical (Fig 1) and the very last 93 residues, which encompass the predicted C-terminal OM-anchoring domain and α-helical region of the molecules, were found to be particularly well-conserved (94.7% identity, Fig 1 and 2). The BPSL1705 ORF is predicted to encode a protein of 148-kDa which, as depicted in Fig 1C, possesses many of the structural features observed in BoaA including two sets of β-roll AIG motifs with the consensus xxG(S/A)(V/I)**AIG**xx(N/A)xAx and several SLST repeats. This high level of sequence and structural similarity between BPSL1705 and BoaA prompted us to designate this *B. pseudomallei *K96243 gene product BoaB.

The *boaB *gene was sequenced from *B. pseudomallei *DD503 and was predicted to encode a protein that is 96.7% identical to BoaB of *B. pseudomallei *K96243. Database searches using NCBI genomic BLAST revealed that the genomes of at least 10 more *B. pseudomallei *strains contain the gene. Overall, the BoaB proteins are highly-conserved (90-99% identity) and characteristics of the ORF from selected strains are shown in Tables 1 and 2 and Fig 2 for comparison purposes. Importantly, database searches also revealed that none of the *B. mallei *isolates available through the NCBI genomic BLAST service have a *boaB *gene. Taken together, these results indicate that BoaB is a highly-conserved *B. pseudomallei*-specific molecule.

### Expression of the *Burkholderia *BoaA and BoaB proteins in *E. coli*

**Figure 3:**
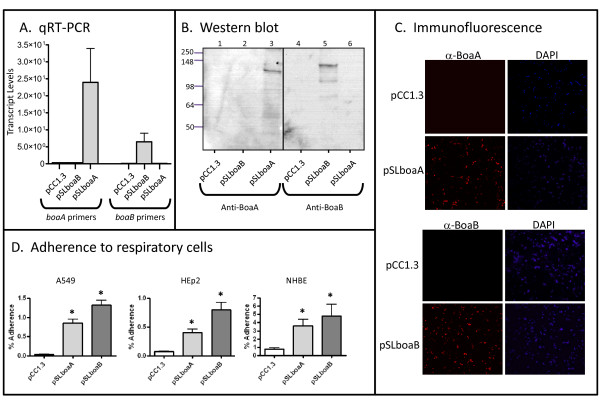
**Analysis of recombinant *E. coli *strains**. Panel A: Total RNA was isolated from *E. coli *strains, reverse-transcribed to cDNA, and the relative levels of *boaA *or *boaB *transcript were determined by qRT-PCR. Each bar represents 4 different samples collected on 2 separate occasions. The Y-axis corresponds to the levels of *boaA *or *boaB *transcript normalized to *recA *and the error bars correspond to the standard error. Negative controls in which the reverse transcriptase enzyme was not added to reaction mixtures were included in all experiments (data not shown). Panel B: Proteins present in Sarkosyl-insoluble OM protein preparations were resolved by SDS-PAGE, transferred to PVDF membranes and analyzed by western blot with antibodies against BoaA (lanes 1-3) or BoaB (lanes 4-6). Lanes 1 & 4, *E. coli *(pCC1.3); lanes 2 & 5, *E. coli *(pSLboaB); lanes 3 & 6, *E. coli *(pSLboaA). MW markers are shown to the left in kilodaltons. Panel C: Non-permeabilized *E. coli *strains were fixed onto glass slides and fluorescently-labeled with DAPI (blue) and with α-BoaA or α-BoaB antibodies (red). Bacteria were visualized by microscopy using a Zeiss LSM 510 Meta confocal system. Representative microscopic fields are shown. Panel D: *E. coli *strains were incubated with A549 and HEp2 cells for 3-hr and with NHBE cultures for 6-hr. Epithelial cells were washed to remove unbound bacteria, lysed, diluted, and spread onto agar plates to enumerate bound bacteria. The results are expressed as the mean percentage (± standard error) of inoculated bacteria adhering to epithelial cells. Asterisks indicate that the increased adherence of the indicated strains, compared to *E. coli *carrying the control plasmid pCC1.3, is statistically significant (P < 0.05). These attachment assays were performed in duplicate on at least 3 separate occasions.

Because of their sequence and structural similarities to known bacterial adhesins, we hypothesized that BoaA and BoaB mediate adherence to human epithelial cells. To test this hypothesis, the *B. mallei *ATCC23344 *boaA *and *B. pseudomallei *DD503 *boaB *genes were cloned into the *E. coli *strain EPI300. This organism does not normally adhere well to human epithelial cells [61,62,66] and therefore provides an appropriate heterologous genetic background for examining the adhesive properties of BoaA and BoaB. To verify gene expression, RNA was prepared from *E. coli *harboring the plasmids pCC1.3 (control), pSLboaA (specifies *B. mallei *ATCC23344 *boaA*) and pSLboaB (specifies *B. pseudomallei *DD503 *boaB*), and analyzed by quantitative Reverse-Transcriptase PCR (qRT-PCR). Fig 3A demonstrates that the *boaA *and *boaB *genes are expressed by recombinant bacteria and that the primers used in these experiments are specific for their corresponding genes. Sarkosyl-insoluble OM proteins were also extracted from *E. coli *cells and analyzed by western blot to ensure production of the *Burkholderia *proteins. Fig 3B shows that α-BoaA antibodies (Abs) react with a band of 130-kDa in the OM of *E. coli *expressing *boaA *(lane 3) whereas Abs against BoaB bind to a 140-kDa antigen in *E. coli *expressing *boaB *(lane 5). These molecular weights (MWs) are consistent with the predicted masses of the gene products (Table 1).

In addition to showing that BoaA and BoaB are associated with the OM by protein separation and western blot, we used immunofluorescent labeling of non-permeabilized *E. coli *cells to demonstrate their display on the bacterial surface. As depicted in Fig 3C, *E. coli *harboring pSLboaA and pSLboaB are labeled by the α-BoaA and α-BoaB Abs, respectively, while recombinant bacteria carrying the control plasmid pCC1.3 are not. Staining of nucleic acids with the fluorescent dye DAPI verified that comparable numbers of bacterial cells were examined (Fig 3C). Quantitative attachment assays revealed that *E. coli *expressing BoaB attach to HEp2 (laryngeal) and A549 (type II pneumocytes) epithelial cell lines at levels 18- and 68-fold greater than bacteria carrying pCC1.3, respectively (Fig 3D). In addition, BoaB expression was found to increase adherence to differentiated primary cultures of normal human bronchial epithelium (NHBE). Under the growth conditions used, NHBE cultures form a pseudostratified epithelium with tight junctions containing both ciliated and non-ciliated cells. This epithelium exhibits transepithelial resistance, mucus secretion, mucociliary activity, and an apical surface not submerged in tissue culture medium, thus representing an environment that is similar to the airway lumen *in vivo *[67-69]. Expression of the *B. mallei *ATCC23344 BoaA protein on the surface of *E. coli *also substantially increased adherence to monolayers of A549 and HEp2 cells and to NHBE cultures. Taken together, these data demonstrate that BoaA and BoaB are OM proteins mediating adherence to epithelial cells of the human respiratory tract.

*B. pseudomallei *and *B. mallei *are facultative intracellular organisms that can invade, survive and replicate in a variety of eukaryotic cells. Moreover, autotransporter adhesins often specify additional biological functions such as invasion [70], biofilm formation [71], survival within host cells [72] and intracellular motility [16]. For these reasons, we measured the ability of *E. coli *expressing BoaA and BoaB to invade epithelial cells as well as their ability to survive within murine macrophages. We also measured the ability of these recombinant strains to form biofilms on the plastic support of tissue culture plates using a crystal violet-based assay. The results of these experiments indicated that neither BoaA nor BoaB substantially increase invasion of epithelial cells, phagocytosis of recombinant bacteria by J774A.1 murine macrophages, survival inside these immune cells, or biofilm formation (data not shown).

### Construction and characterization of *Burkholderia *mutant strains

**Figure 4:**
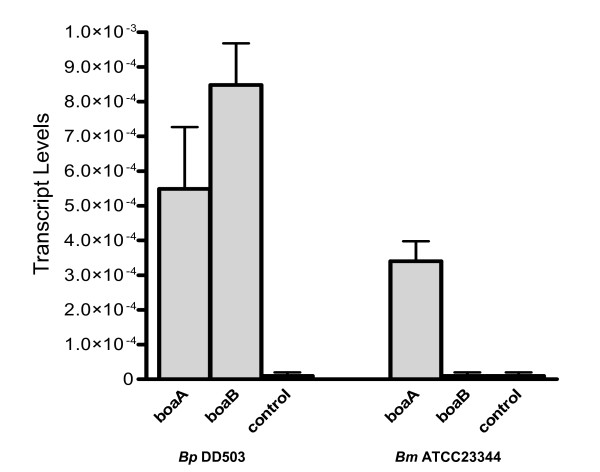
**Quantitative reverse-transcriptase PCR analysis of *B. mallei *and *B. pseudomallei *strains**. Total RNA was isolated from *B. pseudomallei *(*Bp*) DD503 and *B. mallei *(*Bm*) ATCC23344, reverse-transcribed to cDNA, and the relative levels of *boaA *or *boaB *transcript was determined by qRT-PCR. Each bar represents 4 different samples collected on 2 separate occasions. The Y-axis corresponds to levels of *boaA *or *boaB *transcript normalized to *recA *and the error bars correspond to the standard error. A primer set for *Borrelia burgdorferi recA *was used as a control to further demonstrate primer specificity (see bars labeled as control). Of note, negative controls in which the reverse transcriptase enzyme was not added to reaction mixtures were included in all experiments and the results were equivalent to the *Borrelia burgdorferi *controls (data not shown).

To study the functional properties of the *boa *gene products in the native *Burkholderia *background, we constructed isogenic *boaA *mutants of *B. pseudomallei *DD503 and *B. mallei *ATCC23344 as well as an isogenic *boaB *mutant of *B. pseudomallei *DD503. A double mutant strain was also engineered in which inactivated versions of both *boaA *and *boaB *were introduced in the genome of *B. pseudomallei *DD503. Whole cell lysates and sarkosyl-insoluble OM proteins were prepared from these strains and analyzed by western blot to verify lack of BoaA and BoaB expression in the mutants. The α-BoaA and α-BoaB Abs, however, did not react with *Burkholderia *protein preparations (data not shown). In order to determine whether the genes are expressed, total RNA was isolated from *B. pseudomallei *DD503 and *B. mallei *ATCC23344 and the relative transcript levels of *boaA *and *boaB *were assessed by qRT-PCR. Fig 4 shows that *boaA *and *boaB *are expressed by *B. pseudomallei *while *B. mallei *only expresses *boaA*, which is in agreement with database searches revealing that *B. mallei *isolates do not contain a *boaB *gene. The qRT-PCR data also demonstrate that the genes are expressed at very low levels compared to *Burkholderia recA*, which was used to normalize *boaA *and *boaB *transcript levels. These results are consistent with our inability to visualize the proteins by western blot. Other methods such as immunoprecipitation and immunofluorescence labeling also proved unsuccessful at detecting production of BoaA and BoaB by *Burkholderia *strains.

**Figure 5:**
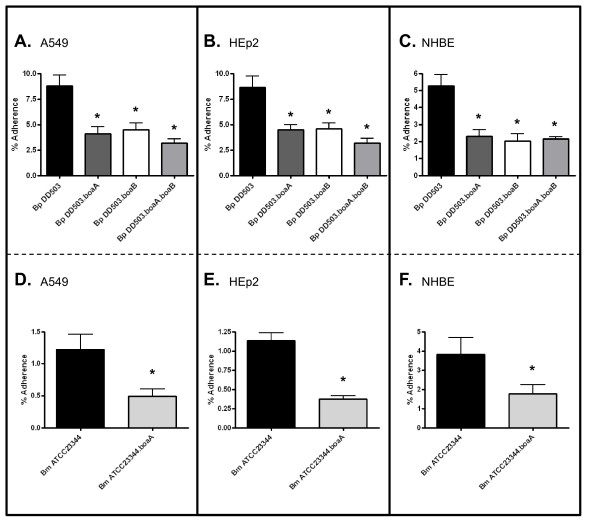
**Adherence of *B. mallei *and *B. pseudomallei *strains to human respiratory epithelial cells**. The effects of *boaA *and *boaB *mutations on the adherence of *B. pseudomallei *(*Bp*) DD503 and *B. mallei *(*Bm*) ATCC23344 to monolayers of A549 (panels A and D) and HEp2 (panels B and E) cells and cultures of NHBE (panels C and F) was measured in duplicate on at least 3 separate occasions. The results are expressed as the mean percentage (± standard error) of inoculated bacteria adhering to epithelial cells. Asterisks indicate that the difference between the adherence of the mutant and that of the parental strain is statistically significant (*P *< 0.05).

Quantitative attachment assays with recombinant bacteria indicated that BoaA or BoaB expression significantly increases the adherence of *E. coli *to monolayers of A549 and HEp2 cells and to NHBE cultures (Fig 3D). We therefore compared the ability of *Burkholderia *parent and *boa *mutant strains to attach to these respiratory cells. As shown in Fig 5A and 5D, inactivation of the *boaA *gene in *B. mallei *ATCC23344 and *B. pseudomallei *DD503 decreases adherence to A549 cells by 60 and 53%, respectively. The *boaA *mutation also caused a 50% reduction in the binding of *B. pseudomallei *to HEp2 monolayers (Fig 5B), and reduced adherence of *B. mallei *to these laryngeal cells by 67% (Fig 5E). Moreover, both *boaA *mutant strains displayed significant impairment in their abilities to attach to NHBE cultures (Fig 5C and 5F). The *boaB *mutation in *B. pseudomallei *DD503 decreased attachment to A549 and HEp2 cells by ~50% (Fig 5A and 5B, respectively) and caused a 62% reduction in adherence to NHBE cultures (Fig 5C). As expected, the double mutant strain DD503.boaA.boaB exhibited significantly lower attachment to epithelial cells compared to the parent strain DD503 (Fig 5A, B, and 5C). The adherence levels of the double mutant, however, did not differ significantly from that of the single mutants in any of the cell types tested. One possible explanation for this apparent lack of synergistic effect is that other adhesins expressed by the double mutant strain DD503.boaA.boaB provide a high background level of adherence. Taken together, these results demonstrate that the *boaA *and *boaB *gene products contribute to the adherence of *B. mallei *and *B. pseudomallei *to epithelial cells of the human respiratory tract.

**Figure 6:**
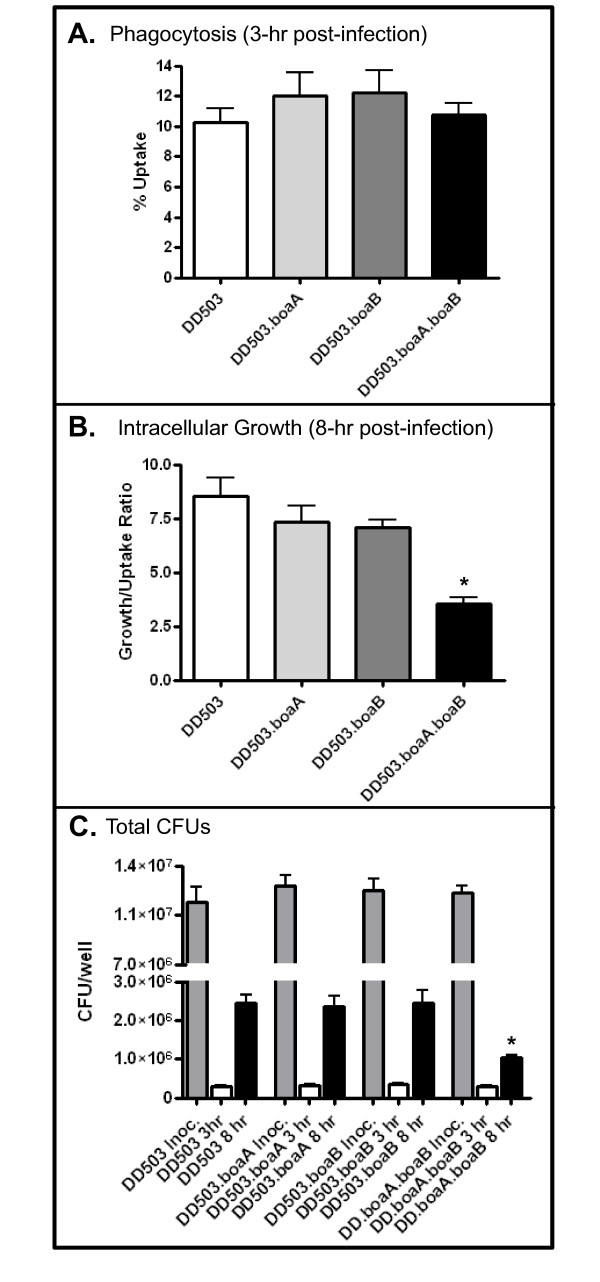
**Uptake and growth of *B. pseudomallei *strains in J774A.1 murine macrophages**. J774A.1 cells (duplicate wells in each of two 24-well tissue culture plates) were infected with *B. pseudomallei *strains at an MOI of 10 and incubated for 1-hr to allow phagocytosis of the organisms. Following incubation, the monolayers were incubated for 2-hr in medium containing gentamicin to kill extracellular bacteria. After gentamicin treatment (*i.e. *3-hr post infection), the wells of one plate were washed, lysed, serially diluted, and spread onto agar plates to determine the number of bacteria phagocytosed by macrophages. The results of this first part of the experiments (*i.e. *bacterial uptake) are shown in panel A and are expressed as the percentage of bacteria (± standard error) used to infect macrophages that were phagocytosed. The wells of the other tissue culture plate inoculated with *B. pseudomallei *strains were washed once, fresh medium without antibiotics was added to wells, and the plate was incubated for an additional 5-hr. Following this incubation (*i.e. *8-hr post-infection), the wells were processed as described above in order to enumerate bacterial numbers. The results of this second part of the experiments (*i.e. *intracellular growth of phagocytosed bacteria) are shown in panel B and are expressed as a growth/uptake ratio (± standard error) obtained by dividing the number of bacteria/well at 8-hr post infection by the number of bacteria/well at the 3-hr post infection time point. These experiments were repeated on at least 3 separate occasions. The asterisk indicates that the difference between the intracellular growth of the double mutant strain DD503.boaA.boaB and that of its parent isolate DD503 is statistically significant (*P *< 0.05). Panel C shows the total number of bacteria in the inoculum (grey bars), the number of phagocytosed bacteria (open bars, 3-hr post infection) and the total number of bacteria/well at the end point of the experiment (black bars, 8-hr post infection).

As previously stated, autotransporter adhesins often specify additional biological functions including survival within host cells [72]. In addition, *B. pseudomallei *and *B. mallei *are facultative intracellular pathogens that are particularly proficient at replicating inside professional phagocytic cells. For these reasons, we measured the ability of our panel of *Burholderia *mutant and parent strains to replicate within J774A.1 murine macrophages. In *B. pseudomallei *DD503, inactivation of the *boa *genes had no effect on phagocytosis of the organism (Fig 6A). Once inside macrophages, the *boaA *(DD503.boaA) and *boaB *(DD503.boaB) single mutants replicated at rates equivalent to that of the progenitor strain DD503 (Fig 6B). However, when both *boaA *and *boaB *genes were disrupted (DD503.boaA.boaB), intracellular growth was diminished by 60% (Fig 6B). To verify that this reduced intracellular fitness was not due to a global growth defect, we measured the growth of strains DD503 and DD503.boaA.boaB in broth as well as in tissue culture medium. We found that both strains grew at equivalent rates under both conditions (data not shown). Interestingly, the double mutant did not exhibit a growth defect in epithelial cells (data not shown). These results suggest a role for the BoaA and BoaB proteins in *B. pseudomallei*'s ability to grow inside professional phagocytes. No defect in uptake or intracellular growth was measured for the *B. mallei *ATCC23344 *boaA *mutant strain (data not shown). It should also be noted that none of the *boa *mutants showed decreased biofilm formation on the plastic support of tissue culture plates nor defects in resistance to the bactericidal activity of normal human serum (data not shown), both biological functions that are also commonly associated with Oca autotransporter adhesins [56,63,73-75].

## Discussion

Autotransporters are involved in various biological traits of Gram-negative bacteria including invasion [70], serum resistance [56,73], phospholipolysis [76,77], cytotoxicity [78], adherence [61,79], biofilm formation [71,80], survival within eukaryotic cells [72] and intracellular motility [16]. These proteins share an N-terminal extracellular passenger domain that specifies the biological activity of the autotransporter and a C-terminus containing several β-strands, which tether the molecule to the OM. Based on the structure of this membrane-anchoring domain, autotransporters can be classified as conventional (contain 12 β-strands) or trimeric (contain 4 β-strands) [65]. One of the best characterized trimeric autotransporters is the *Y. enterocolitica *adhesin YadA. This protein, along with structurally-related adherence proteins such as *M. catarrhalis *Hag and *H. influenzae *Hia, are often referred to as oligomeric coiled-coil adhesins (Oca) [55].

Tiyawisutsri and colleagues previously reported that the published genomic sequences of *B. pseudomallei *K96243 and *B. mallei *ATCC23344 contain several ORFs encoding putative trimeric autotransporters [81]. Of these, only BimA (*i.e*. *B. pseudomallei *and *B. mallei *locus tag numbers BPSS1492 and BMAA0749, respectively) has been functionally characterized and shown to be required for actin-based motility of the organisms inside eukaryotic cells [16,17]. In the present study, we identified the *boaA *ORF based on similarities to the Oca proteins *Y. enterocolitica *YadA and *M. catarrhalis *Hag. Specifically, we searched the genome of *B. mallei *ATCC23344 for gene products specifying N-terminal AIG β-roll motifs, a transporter module containing 4 β-strands, and a YadA-like C-terminal domain (PF03895). We demonstrated that when expressed by *E. coli*, *boaA *increases adherence to the human epithelial cell lines HEp2 (laryngeal cells) and A549 (type II pneumocytes) grown as monolayers in submerged cultures. Though these cell types are relevant to the aerosol route of infection by *B. mallei *and *B. pseudomallei*, they lack important features of the airway mucosa such as cilia and mucociliary activity. The ciliated cells of the respiratory tract and other mucosal membranes keep secretions moving and contribute to preventing colonization by pathogens. For these reasons, we also measured the adherence of *E. coli *expressing BoaA to cultures of normal human bronchial epithelium (NHBE) grown in an air-liquid interface system. These cultures mimic the structure and function of the airway mucosa more accurately as they are fully differentiated, form a pseudostratified epithelium with tight junctions, contain ciliated and mucus-producing goblet cells, and exhibit mucociliary activity [67-69]. Quantitative attachment assays utilizing this culture system revealed that BoaA expression increases adherence to NHBE cultures (Fig 3D).

In addition to showing that BoaA specifies adhesive properties when expressed in the heterologous genetic background of *E. coli*, we determined that disruption of the *boaA *gene in the genome of *B. mallei *ATCC23344 reduces adherence of the organism to monolayers of HEp2 and A549 cells and to NHBE cultures, therefore substantiating the function of BoaA as an adhesin. Database searches using the NCBI genomic BLAST service identified *boaA *in several *B. pseudomallei *and *B. mallei *isolates and we demonstrated that inactivation of *boaA *in the *B. pseudomallei *strain DD503 also decreases attachment to HEp2 laryngeal cells, A549 pneumocytes, and NHBE cultures. Together, our data indicate that BoaA is an adhesin common to *B. mallei *and *B. pseudomallei *and mediates adherence to host cells relevant to pathogenesis by the organisms. These findings are consistent with the recent inclusion of BoaA (*i.e. B. mallei *ATCC23344 and *B. pseudomallei *K96243 locus tag numbers BMAA0649 and BPSS0796, respectively) in the virulome of *B. mallei *and *B. pseudomallei*, which consists of a set of 650 putative virulence genes that are shared by *B. pseudomallei *and *B. mallei *but are not present in five closely-related non-pathogenic *Burkholderia *species [82].

Comparative genomic analyses revealed that several *B. pseudomallei *isolates possess a second Oca-like gene product highly similar to BoaA, which we termed BoaB. The C-terminus of BoaB is strikingly similar to that of BoaA (Fig 2) and the predicted passenger domains of the molecules contain numerous matching serine-rich SLST motifs (Fig 1). The proteins are also functionally related as they mediate adherence to the same types of host cells (Fig 3D and 5). Therefore, it is tempting to speculate that *boaA *and *boaB *are the result of gene duplication. This hypothesis would be consistent with the genomic organization of the genes. In *B. pseudomallei *strains K96243, 1710b, 1655, 576 and MSHR346, the *boaB *gene is located on chromosome 1 while *boaA *is on chromosome 2. Moreover, the *boaB *gene in all these isolates is preceded by two ORFs specifying an invertase and a transposase. These genes may be the remnants of mobile genetic elements possibly involved in gene duplication. Database searches also revealed that *B. mallei *isolates do not possess a *boaB *gene, which was likely lost during evolution of the organism into a host-adapted pathogen. Interestingly, the closely-related bacterium *Burkholderia thailandensis *has been reported by others to bind poorly to epithelial cells [83]. This organism exhibits high genomic similarities to *B. pseudomallei *and *B. mallei *and, like *B. pseudomallei*, is a natural inhabitant of the tropical soil environment. However, *B. thailandensis *is not considered pathogenic to humans or higher animals [84-87]. This difference in virulence can be attributed to the fact that *B. thailandensis *does not produce a capsule [88] and lacks the 650 genes comprising the aforementioned virulome of *B. mallei *and *B. pseudomallei*. Analysis of the published genome of the *B. thailandensis *strain E264 [89] indicated that it contains neither the *boaA *nor the *boaB *gene.

*B. pseudomallei *DD503 and *B. mallei *ATCC23344 do not produce detectable amounts of the BoaA and BoaB proteins under the conditions tested. These results are consistent with qRT-PCR experiments demonstrating that the organisms express very low levels of the *boa *genes relative to the *Burkholderia recA *control (Fig 4). Similar observations were made by Druar and colleagues while studying expression of the *Burkholderia *Type 3 Secretion System-3 (T3SS-3) proteins BipB and BipD [90]. These proteins were not detected in lysates of *B. pseudomallei *or *B. mallei *grown under different conditions, even though the antibodies used in their western blot experiments recognized recombinant forms of BipB and BipD. The authors concluded that these two T3SS-3 molecules must be expressed in detectable amounts only under very specific *in vitro *conditions [90]. Using a *gfp *reporter strain, Burtnick *et al *recently showed that the *B. mallei *Type 6 Secretion System-1 (T6SS-1) gene *tssE *is not expressed at detectable levels when bacteria are grown in LSLB or tissue culture medium, but is expressed upon phagocytosis of the organisms by murine macrophages [49]. The protein preparations tested in our studies were obtained from bacteria cultured on LSLB agar plates at 37°C, conditions which may not be optimal for expression of the BoaA and BoaB proteins. Additionally, Chantratita and colleagues reported that growth of *B. pseudomallei *under various conditions triggers a complex adaptive process altering the expression of surface molecules [91]. This process, termed phenotypic plasticity, was correlated with changes in the morphology of *B. pseudomallei *colonies grown on agar plates and appears to modulate the environmental fitness, as well as virulence, of the organism. Given their surface location and likely role in virulence (*i.e. *adherence to host cells), it is possible that BoaA and BoaB are subject to phenotypic plasticity and are expressed in detectable amounts only under very specific *in vitro *conditions. In concordance, the reduced adherence phenotype of the *boaA *and *boaB *mutant strains suggests increased level of expression of the genes when *Burkholderia *is incubated with epithelial cells. However, efforts to detect protein expression under these conditions (*i.e. *immunofluorescence, immunoprecipitation) have been unsuccessful. Of further note, studies have shown that sera from horses infected with *B. mallei *and sera from melioidosis patients contain antibodies reacting with BoaA (*i.e. B. mallei *ATCC23344 locus tag number BMAA0649) [81] and with BoaB (*i.e. B. pseudomallei *K96243 locus tag number BPLS1705)[92], respectively, which indicates expression of the autotransporters *in vivo*. Determining the conditions and mechanisms that modulate expression of the Boa adhesins, and their influence on the binding of *B. pseudomallei *and *B. mallei *to host surfaces, represent key areas for future study.

Disruption of *boaA *and *boaB *in the *B. pseudomallei *double mutant strain DD503.boaA.boaB was found to have a significant effect on the growth of the organism within murine macrophages (Fig 6B). At present, it is not clear whether BoaA and BoaB play a direct role in intracellular replication. It is possible that the absence of both Boa proteins in the OM of DD503.boaA.boaB affects the proper surface display of another molecule involved in this phenotypic trait. One candidate is LPS, as this molecule was previously shown to play an important role in the ability of *B. pseudomallei *to grow inside host cells [93,94]. *B. pseudomallei *produces multiple T3SS and T6SS that are involved in the intracellular lifestyle of the organism. These specialized secretion apparatuses are used to inject bacterial effector proteins inside host cells where they exert cytopathic effects or manipulate signaling pathways. One important step in this process is the proper docking of bacteria to the host cell to deliver the effectors. Given their roles in adherence, it is possible that the lack of expression of the *boaA *and *boaB *gene products interferes with the delivery of T3SS and/or T6SS cell-altering effectors, which in turn reduces the intracellular fitness of the double mutant strain DD503.boaA.boaB. The *Yersinia pestis *OM adhesin Ail was recently shown to affect delivery of Yop effector proteins to HEp2 cells and macrophages in such a manner [95]. Alternatively, the reduced intracellular growth of the double *boaA boaB *mutant may be due to a greater sensitivity to immune effectors produced by the macrophages. The molecular basis for this phenotype is currently being investigated.

## Conclusion

The present study reports the identification of *B. pseudomallei *and *B. mallei *gene products mediating adherence to epithelial cells. Because of their classification as select agents, there is currently a shortage of tools for genetic studies in *B. pseudomallei *and *B. mallei *(*i.e*. paucity of acceptable antibiotic markers, lack of low copy plasmids suitable for expressing surface proteins), which precluded us from complementing mutants. Our ability to express BoaA and BoaB in *E. coli*, however, conclusively demonstrates that the proteins directly mediate binding to epithelial cells. These results, along with our analyses of the mutant strains, clearly establish that these molecules participate in adherence by *B. pseudomallei *and *B. mallei*. Adherence is an essential step in pathogenesis by most infectious agents because it is necessary for colonization and precedes invasion of host cells by intracellular pathogens. Thus, continued investigation of BoaA and BoaB will yield important information regarding the biology and virulence of these organisms.

## Methods

### Strains, plasmids, tissue culture cell lines and growth conditions

The strains and plasmids used in this study are described in Table 3. *B. pseudomallei *and *B. mallei *were routinely cultured at 37°C using Low Salt Luria Bertani (LSLB) agar (Teknova) supplemented with polymyxin B [PmB] (100 μg/ml for *B. pseudomallei*; 7.5 μg/ml for *B. mallei*), zeocin (100 μg/ml for *B. pseudomallei*; 7.5 μg/ml for *B. mallei*), kanamycin [Kan] (50 μg/ml for *B. pseudomallei*; 5 μg/ml for *B. mallei*), streptomycin [Sm] (used only for *B. pseudomallei*, 1000 μg/ml) and glycerol (used only for *B. mallei*, 5%), where indicated. Plate-grown bacteria (20-hr growth for *B. pseudomallei*; 40-hr growth for *B. mallei*) were used for extraction of Sarkosyl-insoluble outer membrane proteins, preparation of whole cell lysates, RNA isolation, as well as for adherence, invasion, bactericidal, biofilm, and macrophage assays.

**Table 3:** Strains and plasmids

Strain	Description	Reference
*B. pseudomallei*		
DD503	Parental strain; polymyxin B^R ^zeocin^S ^kanamycin^S ^streptomycin^R^	[107]
DD503.boaA	Isogenic *boaA *mutant strain of DD503; polymyxin B^R ^zeocin^R ^kanamycin^S ^streptomycin^R^	This study
DD503.boaB	Isogenic *boaB *mutant strain of DD503; polymyxin B^R ^zeocin^R ^kanamycin^S ^streptomycin^R^	This study
DD503.boaA.boaB	Isogenic *boaA boaB *double mutant strain of DD503; polymyxin B^R ^zeocin^R ^kanamycin^R ^streptomycin^S^	This study

*B. mallei*		
ATCC23344	Wild-type strain; polymyxin B^R ^zeocin^S ^kanamycin^S^	[26]
ATCC23344.boaA	Isogenic *boaA *mutant strain of ATCC23344; polymyxin B^R ^zeocin^R ^kanamycin^S^	This study

*E. coli*		
EPI300	Cloning strain	EPICENTRE^® ^Biotechnologies
S17	Strain used for conjugational transfer of suicide plasmids from *E. coli *to *B. pseudomallei *or *B. mallei*	[108]

Plasmids		
pCC1™	Cloning vector; chloramphenicol resistant (Cm^R^)	EPICENTRE^® ^Biotechnologies
pKAS46	Mobilizable suicide plasmid; kanamycin^R ^and ampicillin^R^	[109]
pCC1.3	pCC1-based plasmid control, does not confer adherence; Cm^R^	[102]
pSLboaA	pCC1 containing the *B. mallei *ATCC23344 *boaA *gene; Cm^R^	This study
pSLboaAZEO	pSLboaA in which a zeocin^R ^marker was introduced near the middle of the *boaA *gene; Cm^R ^and zeocin^R^	This study
pKASboaAZEO	pKAS46 containing the insert from pSLboaAZEO; zeocin^R ^, ampicillin^R ^and kanamycin^R^	This study
pSLboaB	pCC1 containing the *B. pseudomallei *DD503 *boaB *gene; Cm^R^	This study
pSLboaBZEO	pSLboaB in which a zeocin^R ^marker was introduced near the middle of the *boaB *gene; Cm^R ^and zeocin^R^	This study
pKASboaBZEO	pKAS46 containing the insert from pSLboaBZEO; zeocin^R ^, ampicillin^R ^and kanamycin^R^	This study
pKASboaB5'	pKAS46 containing a 0.8-kb insert which corresponds to a region located within the 5' end of the *B. pseudomallei *DD503 *boaB *ORF; ampicillin^R ^and kanamycin^R^	This study
pKASboaB5'Amp^S^	pKASboaB5' in which the ampicillin^R ^marker was removed; ampicillin^S ^and kanamycin^R^	This study
pEM7ZEO	Source of the zeocin^R ^marker; ampicillin^R ^and zeocin^R^	Invitrogen™

*E. coli *was cultured using LSLB containing 15 μg/ml chloramphenicol, 50 μg/ml Kan or 50 μg/ml zeocin, where indicated. For preparation of plasmid DNA, extraction of Sarkosyl-insoluble outer membrane proteins, RNA isolation, immunofluorescence labeling, as well as for adherence, invasion and macrophage assays, recombinant *E. coli *strains were grown in LSLB supplemented with the EPICENTRE^® ^Biotechnologies CopyControl™ Induction Solution as previously reported [96].

The epithelial cell lines HEp2 (human laryngeal epithelium; ATCC CCL-23) and A549 (type II alveolar lung epithelium; ATCC CCL85) were cultured as outlined by others [97] and the murine macrophage cell line J774A.1 (ATCC TIB-67) was grown in DMEM medium (Mediatech, Inc) supplemented with 10% fetal bovine serum (Invitrogen™) at 37°C and in the presence of 7.5% CO_2_. Normal human bronchial epithelium (LONZA) were expanded, cryopreserved and cultured in an air-liquid interface system as previously described [67-69]. Normal human bronchial epithelium (NHBE) were grown on Transwell permeable inserts (Corning) and their apical surfaces were exposed to air for a minimum of 3 weeks prior to use in biological assays to ensure proper cellular differentiation and the development of functional cilia.

### Recombinant DNA methodology

Standard molecular biology techniques were performed as described elsewhere [98]. Genomic DNA was isolated using the Invitrogen™ Easy-DNA™ kit. Plasmid DNA was obtained with the QIAprep Spin Miniprep Kit (Qiagen). The Failsafe™ PCR System (EPICENTRE^® ^Biotechnologies) was used to amplify the 5.5-kb *boaA *gene of *B. mallei *ATCC23344 with primers P1 (5'-TCA GAT GAA CCG CGT TTC CGT ATC-3') and P2 (5'-ACT CAT ACG GCT CGC GCA TAA A-3'). This amplicon was cloned in the vector pCC1™ using the CopyControl™ PCR Cloning Kit (EPICENTRE^® ^Biotechnologies), yielding the plasmid pSLboaA (Table 3). The 5.4-kb *boaA *gene of *B. pseudomallei *DD503 was amplified with P3 (5'-GCT TGC CGC ACG CAA TGG CT-3') and P4 (5'-ATG GCG AGC GCG AAA CAT GGA AA-3') and the purified PCR product was used as a template in sequencing reactions. The 5.9-kb *boaB *gene of *B. pseudomallei *DD503 was generated with the Failsafe™ PCR system using P5 (5'-TCC ATA AAT TCC CGG CGC TTG TTG-3') and P6 (5'-TGT CTC GAC ATC AGC GGT TCA CTT-3'), sequenced, and then cloned in pCC1™ as described above, yielding the plasmid pSLboaB (Table 3). Of note, the inserts of plasmids pSLboaA and pSLboaB were sequenced to verify that PCR did not introduce mutations resulting in amino acid (aa) substitutions in the *boaA *and *boaB *gene products.

### Construction of *boaA *isogenic mutant strains of *B. mallei *and *B. pseudomallei*

A 0.45-kb zeocin^R ^cassette was introduced into a unique *NheI *site located near the middle of the *boaA *ORF in pSLboaA. The resulting construct, designated pSLboaAZEO, was digested with *BamHI *and a 6-kb fragment corresponding to the *boaA *ORF interrupted by the zeocin^R ^marker was excised from an agarose gel, purified with the High Pure PCR Product Purification Kit (Roche Applied Science), and treated with the EPICENTRE^® ^Biotechnologies End-It™ DNA End Repair Kit. This blunt DNA fragment was then subcloned into the *EcoRV *site of the suicide vector pKAS46. The resulting plasmid, pKASboaAZEO, was introduced into the *E. coli *strain S17 by electroporation and subsequently transferred into *B. mallei *ATCC23344 or *B. pseudomallei *DD503 by conjugation as reported by others [99].

Upon conjugation, *B. pseudomallei *colonies were first selected for resistance to PmB (to prevent growth of *E. coli *S17) and zeocin (to select strains containing the disrupted copy of *boaA *in their genome). These putative mutants were then tested for their sensitivity to kanamycin and resistance to streptomycin, which identified strains that did not contain the suicide vector pKAS46 integrated in their genome. Lastly, these PmB^R ^zeocin^R ^Kan^S ^Sm^R ^conjugants were screened by PCR using Platinum^® ^*Pfx *DNA Polymerase (Invitrogen™) with the primers P7 (5'-TTG AGC ACG ACC AAC AGC AAC GTC-3') and P8 (5'-CCA ATG CGG TCG AAT GAT TGC C-3'), which led to the identification of the mutant strain DD503.boaA. These primers yielded a PCR product of 1.3-kb in *B. pseudomallei *DD503 and a larger amplicon of 1.8-kb in the mutant. The primers P9 (5'-TAT CGC AAG GTT TGG AAC AAG GCG-3') and P10 (5'-ACG CCG AAT ACC CTT GAT AGC TG-3') were also used to further confirm gene replacement in the *B. pseudomallei *mutant strain. These primers amplified DNA fragments of 5-kb in the parent strain DD503 and of 5.5-kb in the isogenic *boaA *mutant. After the conjugative transfer of plasmid pKASboaAZEO into the *B. mallei *strain ATCC23344, colonies shown to be PmB^R^, zeocin^R ^and Kan^S ^were screened by PCR with P7 and P8 as described above to identify the mutant strain ATCC23344.boaA. Of note, the *boaA *genes of both isogenic mutant strains DD503.boaA and ATCC23344.boaA were amplified and sequenced in their entirety to verify proper allelic exchange and successful disruption of *boaA*.

### Construction of a *boaB B. pseudomallei *isogenic mutant strain

The plasmid pSLboaB was digested with *NheI *to remove a 162-bp fragment internal to the *boaB *ORF, treated with the End-It™ DNA End Repair Kit and ligated with the 0.45-kb zeocin^R ^marker to yield the construct pSLboaBZEO. This plasmid was digested with *BamHI *and a 6.2-kb fragment, which corresponds to the *boaB *ORF disrupted with the zeocin^R ^cassette, was purified from agarose gel slices, subcloned into the suicide plasmid pKAS46 and introduced into *B. pseudomallei *DD503 by conjugation as described above. Conjugants shown to be PmB^R ^zeocin^R ^Kan^S ^Sm^R ^were screened by PCR using Platinum^® ^*Pfx *DNA Polymerase (Invitrogen™) with primers P11 (5'-AGG TGG CGAC TCA AAT AGA ACC GT-3') and P12 (5'-GTT CGT GTT GTT GGC TAC GGC AAT-3') to identify the mutant strain DD503.boaB. These primers amplified a PCR product of 1.7-kb in *B. pseudomallei *DD503 and of 2.0-kb in the mutant. The primers P13 (5'-AGG TGG CGA CTC AAA TAG AAC CGT-3') and P10 were also used to further confirm gene replacement in the *B. pseudomallei *mutant strain. These primers generated amplicons of 5.2-kb and 5.5-kb in strains DD503 and DD503.boaB, respectively. Additionally, the *boaB *gene of DD503.boaB was amplified and both strands of the PCR product were sequenced to verify allelic exchange.

### Construction of a *B. pseudomallei boaA boaB *double mutant strain

A 0.8-kb PCR product, which corresponds to a region located within the 5'end of the *B. pseudomallei *DD503 *boaB *ORF, was amplified with Platinum^® ^*Pfx *DNA Polymerase (Invitrogen™) using primers P14 (5'-CTC GGG CTC AAT AAC ATG GC-3') and P15 (5'-CGG AAT TCC GGT TCG TGT TGT TGG CT-3'; *EcoRI *site underlined). This amplicon was digested with *EcoR1 *and directionally cloned into the *EcoRV *and *EcoR1 *sites of the suicide vector pKAS46, yielding the plasmid pKASboaB5'. This construct was digested with *ApaLI *to remove a 0.8-kb fragment corresponding to the ampicillin-resistance marker of pKAS46 and the resulting plasmid, pKASboaB5'Amp^S ^, was introduced into the *B. pseudomallei *mutant strain DD503.boaA by conjugation as described above. Conjugants shown to be PmB^R ^zeocin^R ^Kan^R ^Sm^S ^were screened by PCR using the MasterAmp™ Extra-Long PCR kit (EPICENTRE^® ^Biotechnologies) with primers P13 and P10 to identify the mutant strain DD503.boaA.boaB. These primers amplified PCR products of 5.2-kb in *B. pseudomallei *DD503 as well as in the single mutant DD503.boaA, and of 11.0-kb in the double mutant strain DD503.boaA.boaB. These results indicated that the *boaB *gene in DD503.boaA.boaB had been disrupted by integration of the entire pKASboaB5'Amp^S ^plasmid into the genome of *B. pseudomallei*.

### Quantitative reverse-transcriptase PCR (qRT-PCR)

Total RNA was extracted from 10^8 ^bacteria with the RNeasy Kit (Qiagen). One μg of total RNA was treated with RQ1 RNAse-Free DNase (Promega) and reverse transcribed with Improm II™ Reverse transcriptase (Promega) using random hexamers (Invitrogen™) under the manufacturer's recommended conditions. PCR quantification of specific cDNA levels was performed using a LightCycler^® ^(Roche Applied Science) rapid fluorescence temperature cycler as reported elsewhere [100]. Briefly, amplification was performed in a 10 μl final volume containing 50 mM Tris (pH 8.3), 3 mM MgCl_2_, 4.5 μg of bovine serum albumin, 200 μM deoxynucleotide triphosphates, a 1:10,000 dilution of SYBR^® ^Green I (Molecular Probes, Inc.), 1 μM each primer, and 1 unit of Platinum^® ^*Taq *DNA Polymerase (Invitrogen™). Amplification was performed for 40 cycles, with each run consisting of an initial melting at 95°C for 2 minutes, followed by melting, annealing, extension, and acquiring temperatures specific to each primer set. Serial dilutions of a representative template cDNA were amplified using each primer set to create a standard curve. Particular transcript levels in experimental samples were calculated by comparison to the corresponding standard curve. All calculated values for the *boaA *and *boaB *genes are normalized to either the *Burkholderia recA *or *E. coli recA *levels. A primer set for *Borrelia burgdorferi recA *[100] was used as a non-*Burkholderia *control to further demonstrate primer specificity (control in Fig 4). Negative controls in which the reverse transcriptase enzyme was not added to reaction mixtures were included in all experiments (data not shown). The *boa *and *recA *transcripts were amplified from the same sets of samples. The primer sets used were: boaAF (5'-ATC GCG AAC AAT GCG AAC GAT GTC-3') and boaAR (5'-AAG CGA ATA AGC CTG ACC TGC GAT-3'), boaBF (5'-AAT GCC GTA GCC AAC AAC ACG AAC-3') and boaBR (5'-TCG TCG AGT AAA GTT GCG AAC CGT-3'), recABURKF (5'-CAC GAA CTG CCT CGT GAT CTT CAT-3') and recABURKR (5'-AAA TGC CTT CGC CGT ACA GGA TGT-3'), recACOLIF (5'-ATG GCT ATC GAC GAA AAC-3') and recACOLIR (5'-GGT TTT ACC GGA AGA TTC C-3'), and recABbF (5'-GTC GAT CTA TTG TAT TAG ATG AGG CTC TCG-3') and recABbR (5'-GCC AAA GTT CTG CAA CAT TAA CAC CTA AAG-3').

### Nucleotide sequence analyses

PCR products and plasmids were sequenced at the University of Michigan Sequencing Core. Chromatograms were assembled using the Sequencher 4.9 software (Gene Codes Corporation). The nucleotide sequences of the *B. pseudomallei *DD503 *boaA *(EF423807) and *boaB *(EF423808) genes were deposited in GenBank under the indicated accession number.

### Bioinformatic Analyses

Sequence analyses were performed using Vector NTI (Invitrogen) and the various online tools available through the ExPASy Proteomics Server (http://au.expasy.org/). Signal sequence cleavage sites were determined using the SignalP 3.0 server (http://www.cbs.dtu.dk/services/SignalP/). The *B. mallei *ATCC23344 *boaA *gene product (locus tag BMAA0649) was identified by searching the genome of the organism for the presence of a YadA-like C-terminal domain (Pfam database number PF03895) through the NCBI genomic BLAST service using the tblastn and blastp programs (http://www.ncbi.nlm.nih.gov/sutils/genom_table.cgi). The other *boaA *and *boaB *gene products described in this study were identified by using the predicted aa sequence of the *B. mallei *ATCC23344 BoaA protein to search the genomes of the *B. mallei *as well as *B. pseudomallei *strains available through the NCBI genomic BLAST service utilizing the tblastn and blastp programs. Structural features of the Boa proteins (*e.g. *helical regions, β-strands) were identified using the PSIPRED Protein Structure Prediction Server (http://bioinf.cs.ucl.ac.uk/psipred/).

### Epithelial cell adherence assays

Quantitative attachment assays were performed as previously described by our laboratory [61,67]. Monolayers of A549 and HEp2 cells and cultures of NHBE were infected with *B. mallei*, *B. pseudomallei *or recombinant *E. coli *strains at a MOI of 100. Duplicate assays were repeated on at least 3 occasions for each strain, and adherence is expressed as the percentage (± standard error) of bacteria attached to epithelial cells relative to the inoculum. Statistical analyses were performed using the Mann-Whitney test (GraphPad Prism software) and *P *values < 0.05 are reported as statistically significant.

### Biofilm and bactericidal assays

These experiments were performed as previously described [96,101,102]. We used 50% and 25% normal human serum in bactericidal assays with *B. pseudomallei *and *B. mallei*, respectively.

### Macrophage survival assays

Plate-grown bacteria were suspended in 5-ml of sterile PBS supplemented with 0.15% gelatin (PBSG) to a density of 10^9 ^CFU/ml. These suspensions were used to infect two identical sets of duplicate monolayers of J774A.1 cells (10^5 ^cells/well; 24-well tissue culture plate) at an MOI of 10. The inoculated tissue culture plates were centrifuged (5-min, 165 × *g*) and incubated for 1-hr at 37°C, time after which the medium covering the monolayers was replaced with fresh tissue culture medium containing 50 μg/ml gentamicin. After a 2-hr incubation (*i.e. *3-hr post infection), the wells of one tissue culture plate were washed, J774A.1 cells were lysed with a solution containing Saponin, and serial dilutions of the well contents were spread onto agar plates to determine the number of bacteria phagocytosed by the macrophages. The wells of the other tissue culture plate were washed once, fresh medium without antibiotics was added, and the plate was incubated for an additional 5-hr. Following this incubation (*i.e. *8-hr post-infection), the wells were processed as described above in order to enumerate bacteria. These experiments were repeated on at least 3 separate occasions. Statistical analyses were performed using the Mann-Whitney test (GraphPad Prism software) and *P *values < 0.05 are reported as statistically significant.

### Epithelial cell invasion and survival assays

These experiments were performed as described above for macrophage survival assays with some modifications. Specifically, epithelial cells were infected with an MOI of 100. The inoculated tissue culture plates were centrifuged and incubated for 3-hr at 37°C, time after which the medium covering the monolayers was replaced with fresh tissue culture medium containing 50 μg/ml gentamicin. After a 2-hr incubation (*i.e. *5-hr post infection), the wells of one tissue culture plate were washed and processed to enumerate intracellular bacteria as described above. The wells of the other tissue culture plate were washed once, fresh medium without antibiotics was added to wells, and the plate was incubated for an additional 3-hr. Following this incubation (*i.e. *8-hr post-infection), the wells were processed as described above. These experiments were repeated on at least 3 separate occasions. Statistical analyses were performed using the Mann-Whitney test (GraphPad Prism software) and *P *values < 0.05 are reported as statistically significant

### Protein preparations, western blot, and antibody production

Sarkosyl-insoluble OM proteins were obtained as previously described by Carlone *et al *[103]. The methods used to prepare whole cell lysates and perform western blot experiments are described elsewhere [61,62,67,104,105]. To obtain antibodies directed against BoaA, the peptide PEPA (NYLGGLFGFGPQTSMANWGDSSN) was synthesized and conjugated to maleimide-activated keyhole limpet hemocyanin (mcKLH, Thermo Scientific) under the manufacturer's recommended conditions. The sequence of PEPA corresponds to residues 78-100 of *B. pseudomallei *DD503 BoaA and encompasses aa 79-101 of *B. mallei *ATCC23344 BoaA (underlined residues in the PEPA sequence being perfectly conserved). The mcKLH-PEPA conjugate was emulsified in Freund's adjuvants and used to immunize female BALB/c mice as previously reported [106]. BoaB-specific antibodies were obtained by immunizing mice with mcKLH conjugated to the synthetic peptide PEPB (GWLLGTTSQTTDPGPLYPGPGAENN), which specifies aa 131-155 of *B. pseudomallei *DD503 BoaB. These animal studies were performed in compliance with institutional, as well as governmental, rules and regulations.

### Immunofluorescence labeling of *E. coli *and microscopy

Plate-grown bacteria were suspended in 5-ml of sterile PBSG to a density of 10^8 ^CFU/ml. Portions of these suspensions were spotted onto glass slides and dried using a warming plate. The slides were fixed with PBSG supplemented with 4% paraformaldehyde for 30-min at room temperature, washed with PBS supplemented with 0.05% Tween 20 (PBST), and blocked overnight at 4°C using PBST supplemented with 10% goat serum (SIGMA-ALDRICH^®^). Next, bacteria were probed for 1-hr at room temperature with murine α-BoaA or α-BoaB antibodies diluted (1:200) in PBST supplemented with 10% goat serum. After this incubation, the slides were washed with PBST to remove unbound antibodies and incubated for 30-min at room temperature with a goat α-mouse antibody labeled with Alexa Fluor^® ^546 (Molecular Probes, Inc) and diluted (1:400) in PBST supplemented with 10% goat serum. Following this incubation, the slides were washed with PBST to remove unbound antibody and bacterial cells were stained using the nucleic acid dye DAPI (Molecular Probes, Inc). Slides were mounted with SlowFade^® ^reagent (Invitrogen™) and examined by microscopy using a Zeiss LSM 510 Meta confocal system.

## Abbreviations

OM: outer membrane; aa: amino acid; ORF: open reading frame; Oca: oligomeric coiled-coil adhesin; MW: molecular weight; CFU: colony forming units; PmB: polymyxin B; Kan: kanamycin; Sm: streptomycin; nt: nucleotide; qRT-PCR: quantitative reverse-transcriptase PCR; cDNA: complementary DNA.

## Authors' contributions

RB helped conceive the study, participated in its design and coordination, performed most of the experiments involving live *B. pseudomallei *and *B. mallei*, and helped with redaction of the manuscript. SL performed several of the experiments involving live *B. pseudomallei *and *B. mallei*. JJL carried out the qRT-PCR experiments. WG carried out some of the macrophage survival assays with *B. pseudomallei *and helped with redaction of the manuscript. RMW contributed to the qRT-PCR experiments, participated in the conception and design of the study. RJH participated in generating antibodies against BoaA and BoaB. DEW provided the strains *B. pseudomallei *DD503, *B. mallei *ATCC23344, and *E. coli *S17, also participated in the design of the study. ERL conceived the study, participated in its design and coordination, performed experiments involving live *B. pseudomallei *and *B. mallei*, and helped with redaction of the manuscript. All authors read and approved the final manuscript.
